# Patient and public involvement and engagement in methodology research: process, experiences, and recommendations from the SPIRIT- and CONSORT-Surrogate project

**DOI:** 10.1186/s40900-025-00807-y

**Published:** 2025-12-04

**Authors:** Anthony Muchai Manyara, Derek Stewart, Sarah Markham, Andrew Worrall, Ray Harris, Philippa Davies, Christopher J. Weir, Amber E. Young, Jane Blazeby, Nancy J. Butcher, Sylwia Bujkiewicz, An-Wen Chan, Dalia Dawoud, Martin Offringa, Mario Ouwens, Gary S. Collins, Joseph S. Ross, Rod S. Taylor, Oriana Ciani

**Affiliations:** 1 MRC/CSO Social and Public Health Sciences Unit, School of Health and Wellbeing, University of Glasgow, Glasgow, UK; 2 Global Health and Ageing Research Unit, Bristol Medical School, University of Bristol, Bristol, UK; 3 Patient and Public Involvement Partner, Nottingham, UK; 4 Department of Biostatistics & Health Informatics, Institute of Psychiatry, Psychology & Neuroscience (IoPPN), King’s College London, London, UK; 5 Patient and Public Involvement partner, Staffordshire, UK; 6 Patient and Public Involvement partner, London, UK; 7 Population Health Sciences, Bristol Medical School, University of Bristol, Bristol, UK; 8 Edinburgh Clinical Trials Unit, Usher Institute, University of Edinburgh, Edinburgh, UK; 9 Bristol NIHR Biomedical Research Centre, Bristol, UK; 10 University Hospitals Bristol and Weston NHS Foundation Trust, Bristol, UK; 11 Child Health Evaluative Sciences, The Hospital for Sick Children Research Institute, Toronto, Canada; 12 Department of Psychiatry, University of Toronto, Toronto, Canada; 13 Biostatistics Research Group, Department of Population Health Sciences, University of Leicester, Leicester, UK; 14 Department of Medicine, Women’s College Research Institute, University of Toronto, Toronto, Canada; 15 Department of Medicine, University of Toronto, Toronto, Canada; 16 Faculty of Pharmacy, Cairo University, Cairo, Egypt; 17 Department of Paediatrics, University of Toronto, Toronto, Canada; 18 AstraZeneca, Mölndal, Sweden; 19 Department of Applied Health Sciences, School of Health Sciences, College of Medicine and Health, University of Birmingham, Birmingham, UK; 20 NIHR Birmingham Biomedical Research Centre, University Hospitals Birmingham NHS Foundation Trust and University of Birmingham, Birmingham, Birmingham, UK; 21 Department of Health Policy and Management, Yale School of Public Health, New Haven, CT USA; 22 Section of General Medicine, Department of Internal Medicine, Yale School of Medicine, New Haven, CT USA; 23 Robertson Centre for Biostatistics, School of Health and Well Being, University of Glasgow, Glasgow, UK; 24 Centre for Research on Health and Social Care Management, SDA Bocconi School of Management, Milan, Italy

**Keywords:** PPIE, Methodology research, Reporting guidelines, Patient and public involvement

## Abstract

**Background:**

While there are increasing calls for Public and Patient Involvement and Engagement (PPIE) in methodology research, including the development of reporting guidelines, practices continue to emerge. This paper reports on the process, experiences, reflections, and recommendations of both the PPIE partners and other researchers participating in the development of (Standard Protocol Items: Recommendations for Interventional Trials (SPIRIT) and Consolidated Standards of Reporting Trials (CONSORT)-Surrogate reporting guidelines.

**Methods:**

Development of the SPIRIT- and CONSORT-Surrogate guidelines involved four phases: (1) literature reviews; (2) an e-Delphi survey; (3) a consensus meeting, and (4) knowledge translation. PPIE was integrated in Phases 2, 3 and 4. An encompassing budgeted PPIE strategy detailing involvement in all project phases was prepared and implemented by researchers and PPIE partners. Implementation included a learning workshop (attended by 19 PPIE partners) to build PPIE partners’ capacity and confidence to participate in the e-Delphi survey (Phase 2) and the invitation of four PPIE partners to the consensus meeting (Phase 3). Experiences and reflections of PPIE in the project, based on feedback surveys from PPIE partners participating in the project and reflective notes from meetings, were used to formulate recommendations.

**Results:**

In total, 19 PPIE partners took part in the e-Delphi survey (Phase 2), four joined the consensus meeting (Phase 3), and consequently co-authored the guidelines and contributed to the development of an educational animation video (Phase 4). Partners felt that facilitators for involvement in Phase 2 included a learning workshop, financial compensation, support during e-Delphi survey participation (such as a glossary and help texts) and for Phase 3, the main facilitator was allowing partners to contribute first during the consensus meeting. The PPIE partners who joined the consensus meeting (Phase 3) presented the patient perspective; reminded researchers of why the project was important; helped with clarification of issues; corrected grammar; suggested strategies to disseminate and implement the extensions; and created humour. Reflecting on the involvement, both the PPIE partners and researchers felt it was valuable to the project.

**Conclusions:**

Based on the experiences, we make six recommendations for integrating PPIE in projects to develop reporting guidelines: involve early; involve with a plan and layered approach; involve meaningfully in a genuine way; involve with support and in safe spaces; involve with reflection and feedback; and involve with a budget to compensate for time and effort.

**Supplementary Information:**

The online version contains supplementary material available at 10.1186/s40900-025-00807-y.

## Abstract (plain language summary)

This study looked at how patients and the public can be helpfully included in creating guidelines for reporting medical research. In the past, these research guidelines have been created only by scientists; however, involving patients and the general public is increasingly viewed as helpful for everyone in helping to ground guidelines in reality.

The researchers created a detailed plan explaining exactly how people would be involved, particularly in the two main activities: a survey to gather opinions and a meeting to discuss the final decisions on the guidelines.

To prepare patients and members of the public, researchers held a workshop attended by 19 people, most of whom then participated in the survey. Four of those who participated in the survey joined a meeting to finalise the guidelines, actively contributing patient viewpoints. These partners became co-authors of the final guidelines and helped create an educational video explaining the guidelines’ importance.

The involvement of patients and members of the public was successful because of supportive measures like preparatory workshops. Both researchers and patient partners agreed that the involvement was helpful and improved the final guidelines.

Based on their experience, the researchers suggest six practices: involve patients from the beginning; create a clear involvement plan; ensure participation is valuable; provide enough support and create a welcoming environment; regularly gather feedback on the process; and budget well to pay for participants’ time fairly.

**Supplementary Information:**

The online version contains supplementary material available at 10.1186/s40900-025-00807-y.

## Introduction

Patient and public involvement (PPIE) is universally accepted as having a positive impact on research. The National Institute for Health Research (NIHR) defines PPIE in research as “research being carried out ‘with’ or ‘by’ members of the public rather than ‘to’, ‘about’ or ‘for’ them” [1] Randomised controlled trials (referred to as trials hereafter) are important research studies that provide robust evidence to evaluate safety and effectiveness of health interventions. The experiences and impact of PPI in trials are well-documented. First, implementation of PPI in trials has involved various tasks, including membership in trial steering committees and advisory groups, conceptualisation of research questions, ethical review, agenda setting, protocol development, piloting, and dissemination of findings [2, 3]. Furthermore, based on combined evidence from 27 reviews, barriers to PPI in trial design included non-prioritisation of meaningful involvement, insufficient time, difficulties with recruiting PPI partners and practical methods of involvement, while facilitators included respectful inclusion, availability of resources (time, funding), training, and flexibility [3]. The impact of PPI in trials has been extensive and has included: improvements in design and framing of research questions; participant enrolment (increasing the odds of enrolment by 16%)[4]; increased motivation for researchers to conduct or continue with trials; positive impacts of PPI on funding and prioritisation of patient-relevant outcomes; better interpretation and dissemination of findings [2, 3].

Apart from involvement in trial design, conduct, and dissemination of findings, there are increasing calls for PPIE in trial methodology research (research into methods used in design, conduct, and reporting of trials [5]). For example, PPIE in trials methodology was one of the research topics suggested in a Delphi survey aimed at setting a methodology research agenda and involving 41 UK Clinical Trials Units directors [5]. Furthermore, Biggane, Olsen, and Williamson noted that PPI can contribute to the quality, integrity, and value of methodological research, but can be challenging to meaningfully include PPIE in some aspects of such research as outputs, unlike intervention research, are not obvious to patients and the public [6]. In 2019, the joint Medical Research Council and National Institute for Health Research Trials Methodology Research Partnership (TMRP) funded a workshop to initiate dialogue with stakeholders on how to effectively include PPI in trial methodology research [7]. The workshop report acknowledges the value of PPIE in trials methodology research but notes that such involvement may need a steep learning curve, and there is a need for researchers to give thought to how PPIE can be integrated in such research [7].

A key aspect of methodology research is the development and implementation of reporting guidelines [8]. Two commonly used guidelines in trials developed through methodology research to improve transparency and reproducibility in clinical trials are: SPIRIT (Standard Protocol Items: Recommendations for Interventional Trials) 2013 statement, a 33-item checklist used to report trial protocols [9]; and CONSORT (Consolidated Standards of Reporting Trials) 2010 statement: a 25-item checklist used to report completed trials [10]. These guidelines are not always sufficient to guide the complete reporting in all types of trials. Therefore, the SPIRIT and CONSORT guidelines are often extended for certain types of trials, i.e., checklist items are modified, or new items are added to original checklists. In 2021, the UK Medical Research Council funded us (a multidisciplinary team of researchers and stakeholders) to extend the SPIRIT and CONSORT guidelines for trials that use surrogate endpoints as the main outcomes [11] which have now been published [12, 13]. Surrogate endpoints are substitutes and predictors of target outcomes, i.e., outcomes that are of importance or benefit to stakeholders such as patients [14, 15]. For example, blood pressure reduction is a surrogate endpoint for stroke, heart attack, and death [13]. While using surrogate endpoints can reduce trial time and cost, allowing faster evaluation and roll-out of interventions [14], there are concerns about their use, given past experiences with trials that used misleading surrogate endpoints, leading to approval of interventions resulting in more harm than health benefit [15–18].

These concerns make surrogate endpoint use very relevant to patients and the public. A survey among diabetes patients reported that the majority preferred the use of patient-relevant outcomes (diabetes-related complications) rather than surrogate endpoints (blood glucose measures) as the main outcomes in trials [19]. Furthermore, the recent Aducanumab controversy [20] (i.e. the US Food and Drug Administration (FDA) approval of Alzheimer’s disease treatment based on a surrogate endpoint rather than a patient-relevant outcome) may have lowered the participants’ willingness to take part in preclinical Alzheimer’s disease trials in those aware of the FDA decision [21].

Acknowledging the relevance of patients and the public in surrogate endpoint use and reporting, we aimed to meaningfully integrate PPIE partners in all project phases (described in detail in the Methods section below): literature review, e-Delphi survey, consensus meeting, and knowledge translation. Our intention was to explore how we might actively involve patients and the public in reviewing and developing these guidelines around one specific aspect: surrogate outcomes. However, the EQUATOR (Enhancing the QUAlity and Transparency Of health Research) Network’s guidance for developing a health research reporting guideline does not have specific guidance on PPIE [22]. Nevertheless, there has been emerging literature of late on PPIE in methodological research, including PPIE in the development of reporting guidelines for systematic review of outcome measurement instruments [23]. Consequently, using the SPIRIT-Surrogate and CONSORT-Surrogate as a case study, we aim to describe the process and activities aimed at PPIE in this project; experiences of PPIE partners who participated; our reflections on the process and experiences; and recommendations on how to integrate PPIE in methodology research. We were keen to actively involve patient/public partners in this project as a means of mutually learning from each other’s experiences and knowledge to improve our understanding of the relevance and meaningfulness of surrogate endpoints and reporting guidelines. PPIE in this project entailed the involvement of patients as participants and partners, including their contributions to the co-authorship of project outputs.

## Methods

### Overall project methodology

**Fig. 1:**
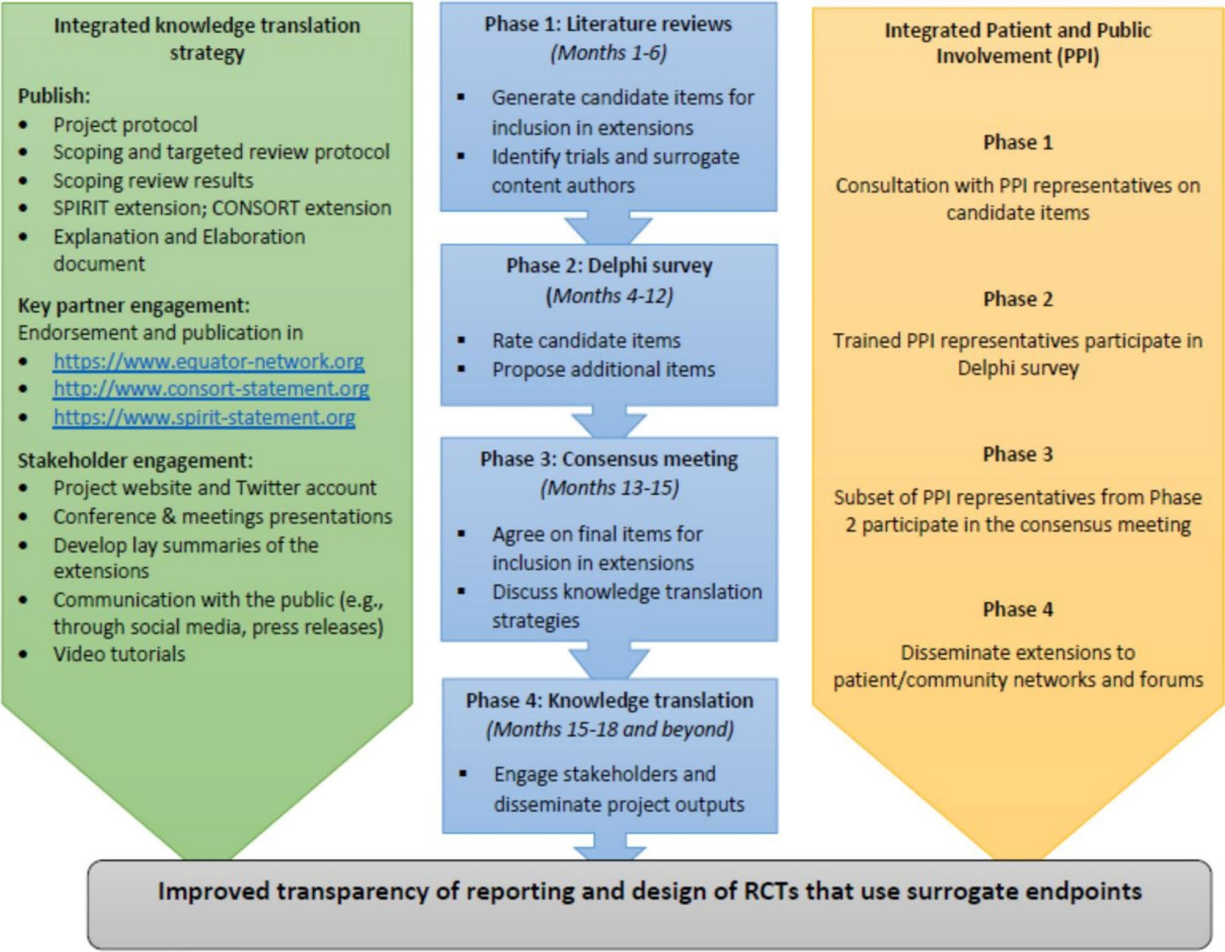
Phases in the development of SPIRIT-and CONSORT-Surrogate guidelines with PPIE integration

Development of SPIRIT- and CONSORT-Surrogate extensions followed the EQUATOR-recommended steps of developing a health reporting guideline [22]. It involved four phases: Phase 1 (Literature reviews) to generate candidate items (i.e. items to be voted for inclusion in final guidelines) to be included in extensions and identify potential participants for the second phase; Phase 2 (Delphi survey) to rate candidate items and allow for suggestion of additional items; Phase 3 (Consensus meeting) to finalise on items to be included in extensions; and Phase 4 (Knowledge translation) to disseminate and implement the extensions. The project and literature review protocols have been published and provide more details on the methods used in the four phases [11, 24], see Fig. 1.

The literature review search was conducted between March and May 2022, and after screening, 90 documents were selected for inclusion. Data on definitions, limitations, acceptability, and guidance were extracted and used to generate 17 potential trial reporting items. Following a project team discussion, 13 items were selected for rating in the e-Delphi survey. Details on the literature review protocol have been published elsewhere [24] as are the findings, including the 17 generated items [25].

A two-round e-Delphi survey using a 9-point Likert scale (1–3: not important; 4–6: important but not critical; 7–9: critical) on the DelphiManager software (version 5.0), maintained by the COMET (Core Outcome Measures in Effectiveness Trials) initiative, http://www.comet-initiative.org/delphimanager/ was conducted between 24th August and 11th December 2022 after piloting. Participants were identified through various strategies, including contacting authors of relevant articles from literature reviews, utilising social media, and calls for participation through professional organisations and networks. A total of 195 and 176 rated the items in rounds one and two, respectively. Participants represented 30 countries and various stakeholders, including trial investigators, trial methodologists, clinicians and allied health professionals, surrogate content experts, journal editors, patient and public partners, regulators, and payers/health technology assessment experts. Consensus thresholds for inclusion of items were: ≥70% score of 7–9 and < 15% score of 1–3; consensus for exclusion: ≥70% score of 1–3 and < 15% score of 7–9. More details, including results of the Delphi surveys, have been published elsewhere [12, 13]. A total of 19 PPIE partners participated in the e-Delphi survey. The 19 PPIE partners represented 9.7% of all participants in the e-Delphi round one and were comparable to the representation of other stakeholder groups such as health technology assessment experts (*n* = 20, 10.3%), clinical guideline/core outcome set developers (*n* = 21, 10.8%), research funding board members (*n* = 17, 8.7%); and higher than trial managers (*n* = 13. 6.7%), ethics committee members (*n* = 11, 5.6%), surrogate content experts (*n* = 13. 6.7%), and regulatory assessors (*n* = 8, 4.1%)[12, 13].

A hybrid consensus meeting (combined face-to-face and virtual meeting to agree on final guidelines items) was held on 13th and 14th March 2023 at the University of Glasgow, Glasgow, UK and via Zoom. Participants included project team members (*n* = 13) and a subset of participants from the e-Delphi survey (*n* = 20). The items (*n* = 4) that did not reach consensus in the e-Delphi survey were discussed and voted on (using www.mentimeter.com). Consensus was predefined as ≥ 70% voting to include or exclude an item. Detailed results of this phase have been published elsewhere [12, 13]. Four PPIE partners participated in the consensus meeting, translating to 12.1% of all participants. This proportion compared to the representation of other stakeholders (*n* = 5 each, 15.2%) – clinical guideline/core outcome set developers, surrogate content experts, health technology assessment experts and epidemiologists – and higher than other stakeholder groups: trial managers (*n* = 2, 6.1%) and regulatory assessors (*n* = 1, 3.0%) [12, 13].

Knowledge translation has been an ongoing process and included raising awareness of the project and its findings through publications [26–35], piloting of the developed guidelines, publication of the main findings [12, 13, 15] and presentations in conferences and meetings. Similar to the consensus meeting, four PPIE (11.1% of all 36 co-authors) were co-authors of the main findings.

### Integration of PPIE

We aimed to integrate PPIE in all four project phases. In the literature reviews, we aimed to share the literature review findings with partners to seek insights on preliminary findings consistent with the consultation phase of the literature review methodology. However, this exercise was not possible, and we reflect on this in the discussion. For the second phase, we organised a learning workshop for PPIE partners to support them in taking part in the e-Delphi survey. The learning workshop was advertised via social media, PPIE networks and by PPIE partners to their peers. It targeted those with some prior knowledge and understanding of trials. The target sample was 20 PPIE partners. A sample of 20 participants for a stakeholder group is sufficient in multistakeholder Delphi surveys [36]. For the third phase, four PPIE partners who helped in the organisation of the learning workshop and/or took part in the survey were invited to be delegates in the consensus meeting. For knowledge translation (Phase 4), we aimed to disseminate the project findings in various community and patient forums.

### Data sources and analysis

The results of this article are divided into four themes: process, experiences, reflections, and recommendations. Descriptive accounts of the process theme are based on the funding application, project meeting notes, email correspondence, and a PPIE strategy developed at the start of the project. The experience theme is based on three online feedback surveys (pre-learning workshop [11th -15th August 2022], post-learning workshop [18th -29th August 2022], post-Delphi survey [13th – 24th February 2023]) and notes from meetings with PPIE partners who took part in the consensus meeting [27th February 2023 and 19th April 2023]. The feedback surveys were developed and piloted by a researcher (AMM) and a PPIE partner (DS). The surveys were conducted using Microsoft Forms and involved the use of multiple-choice questions, Likert scales and free-text boxes to document PPIE knowledge, attitudes, confidence, and experiences, see Supplementary File 1. In addition to documenting experiences of e-Delphi survey participation, the post-Delphi survey was used to evaluate PPIE in the project using modified questions from the public and patient engagement evaluation tool (PPEET) [37]. The reflection accounts are drawn from coauthors’ reflective notes (AMM, DS) and discussions between the coauthors. Finally, the recommendations are based on a synthesis of the process, experiences, and reflection findings presented in this article.

Quantitative findings from surveys are presented in counts and percentages from results generated in Microsoft Forms. Qualitative data from surveys are recorded in a Microsoft Excel sheet and synthesised using a simple form of thematic analysis [38] i.e., similar comments grouped into themes (broad meanings which emerged within the data) by AMM in consultation with DS.

This paper is reported using the Guidance for Reporting Involvement of Patients and the Public (GRIPP2) [39] Long Form, see Supplementary File 2, and Short Form, see Table 1.

**Table 1:** The GRIPP2 short form

Section and topic	Item	Reported on section
1: Aim	Report the aim of PPIE in the study	Introduction; Methods
2: Methods	Provide a clear description of the methods used for PPIE in the study	Methods
3: Study results	Outcomes—Report the results of PPIE in the study, including both positive and negative outcomes	Results
4: Discussion and conclusions	Outcomes—Comment on the extent to which PPIE influenced the study overall. Describe positive and negative effects	Discussion
5: Reflections/critical perspective	Comment critically on the study, reflecting on the things that went well and those that did not, so others can learn from this experience	Results (Reflections)

PPIE = patient and public involvement

## Results

### Process

Table 2 summarises the process of PPIE in the project, including intended and actual involvement or implementation. PPIE started at the conceptualisation stage with DS (renowned international PPIE partner and thought leader), who was a grant co-applicant and led PPIE in the project. After the project started, two other PPIE partners (AW, SM) with experience in trial methodology were invited to help with the implementation of PPIE activities. We wanted to plan an integrated approach that would help improve the guidelines and our own understanding of where PPIE could add value and avoid tokenism. Consequently, a PPIE strategy (see Supplementary File 3), with a budget detailing involvement in all project phases, was prepared by a researcher (AMM) and the PPIE lead (DS). One of the main forms of involvement was through participation in the e-Delphi survey to rate items that would be included in the reporting guidelines. To support meaningful participation in the survey, a two-hour virtual learning workshop was organised by three PPIE partners (DS, AW, SM) and two researchers (AMM, RST). The workshop, held on 15th August 2022, was an interactive session attended by 19 PPIE partners (18 from the UK and one from Canada) who were introduced to surrogate outcomes, trial reporting, and Delphi surveys. The workshop attendees were encouraged to take part in the e-Delphi survey, and the majority who took part in a feedback survey (*n* = 13/15, 87%) reported having participated.

Four PPIE partners were invited to join the hybrid consensus meeting as delegates (one physically and three virtually): they were given an opportunity to contribute before other delegates, and they actively took part in discussions and voting. With reporting guidelines published and as knowledge translation activities intensify, we continue to explore ways of reaching patients and the public. Implementation of the PPIE activities was made possible by costs attached to the project grant (from the UK Medical Research Council) and channelling savings made from the use of a more cost-effective e-Delphi survey platform to the PPIE implementation budget.

**Table 2:** Process of PPIE in the SPIRIT|CONSORT-SURROGATE project included intended and actual involvement of implementation of PPIE activities

Project phases/PPIE activities or requirements	Intended involvement/implementation	Actual involvement/activity implementation
**Project conceptualisation and team constitution**	• To include a PPIE partner as part of the project Executive Committee	• DS was included in the funding application as a member of the Executive Committee. *See involvement of DS in other project phases/activities beyond funding application*.
**Funding**	• Have a budget for PPIE costs in the project grant	• Grant application specified PPIE costs as directly incurred costs. • Savings made from the use of a more cost-effective Delphi survey platform were channelled to the implementation of PPIE.
**Project management**	• As per the funding application, PPIE representative (DS) to be a member of the Executive Committee, which oversees the project and meets quarterly	• After the project started, DS was requested to join the Project Management Group, which had the overall role of delivering the project and met every six weeks
**Roadmap for PPIE in the project**	• Develop a strategy for the integration of PPIE in all project phases	• PPIE strategy developed by DS and AMM listing involvement in the four project phases and an implementation budget
**PPIE advisory group**	• DS to take the role of project PPIE lead and therefore identify 3–5 PPIE partners who would help with the implementation of PPIE strategy	• Two PPIE partners (AW, SM) joined the team and helped in the organisation of the learning workshop and subsequent advisory roles on PPIE in the project*
**Phase 1: Literature reviews**	• PPIE partners to be consulted on synthesised literature review findings, and they comment on language, relevance, and any additional issues not identified by review	• This was not achieved due to time constraints. We note this as a limitation in the discussion. • The PPIE lead (DS) was a co-author in the literature review publication
**Phase 2: Delphi survey**		
Sample size	• Target 20 PPIE partners to participate in the Delphi survey.	• 19 PPIE partners participated in the Delphi survey.
Learning workshop	• Acknowledgement of the need to build confidence of PPIE partners to meaningfully participate in the Delphi survey, hence plan to have a learning workshop	• A 2-hour learning workshop was held on 15th August 2022 and attended by 19 PPIE partners*. • It was not mandatory for the partners who attended the workshop to participate in the Delphi survey, but they were encouraged to participate, and most participated
**Phase 3: Consensus meeting**		
Sample size	• 2–4 PPIE partners who completed the Delphi survey to be picked as consensus meeting delegates	• Four PPIE partners (DS, AW, SM, RH) attended the hybrid consensus meeting, with DS attending physically.
PPIE consensus meeting pre-meet	• A meeting to clarify anything needed for the consensus meeting	• AMM and RST hosted the four PPIE partner consensus meeting delegates for a one-hour virtual meeting to go through the consensus meeting structure and listen to any concerns
PPIE in consensus meeting	• DS attends the consensus meeting co-chairs meeting • Co-chairs acknowledge the need for deliberate hearing of the patient voice in the meeting and for PPIE partners to have the first opportunity to speak during discussions	• Consensus Meeting co-chairs gave PPIE partners the opportunity to speak before opening the discussion to other delegates. • PPIE partners were actively involved in discussion (oral and Zoom chat) and voting.
**Phase 4: Knowledge Translation**		
Project webpage	• Lay summary on the project webpage	• Project webpage has a PPIE subpage with a lay summary of the project
Updates on project progress	• Delphi participants, including PPIE partners, to be informed of the project progress after Delphi survey completion	• Email sent to all Delphi participants updating on project progress on • Separate email sent to all PPIE partners who attended the learning workshop, informing them on project progress
Dissemination of project outputs	• Explore various ways of disseminating main project outputs (extensions) to patients and the public platforms, such as through lay summaries, blogs and webinars	• This continues to be actualised post publication of the extensions. • PPIE partners reviewed a script in the production animated video aimed at educating the public about surrogate endpoints, https://youtu.be/6upTJHdRHpg?si=LAGvrNW4e8Ideg2o. • Webinar organised by Statisticians in the Pharmaceutical Industry (PSI) to disseminate to the extensions involved one of the PPIE partners (RH) presenting the patient and public perspective.
PPIE debrief /evaluation	• Hold a virtual one-hour meeting with 10 PPIE partners to evaluate, reflect and learn from the experiences of PPIE in the project	• Instead of a single project debrief meeting, we used short feedback surveys to capture experiences during PPIE implementation: pre-and post-learning workshop feedback surveys; and post-Delphi feedback survey to understand experiences of participating in the Delphi survey and evaluation of PPIE. • The four PPIE partners (DS, AW, SM, RH) who attended the consensus meeting met for one hour to deliberate on their experiences*.
Co-authoring of extension manuscripts	• PPIE partners who attended the consensus meeting would co-author the two extensions	• PPIE partners (DS, AW, SM, RH) invited to review and edit manuscripts as co-authors [12, 13] • Our lack of published exemplars on one of the extension items (on informing participants on the use of surrogate endpoints) meant that PPIE partners helped with conceptualising exemplars of reporting this item, including how to implement it in the participant information leaflet.
PPIE experiences in the project	• Publish on PPIE in the project	• Achieved through this publication

*Activities with compensation

### Experiences

Generally, PPIE partners who took part in the e-Delphi survey or consensus meeting felt their engagement with the project was valuable. The majority were satisfied with how they were engaged in the project (13/15, 87%); felt that the engagement was a good use of their time (12/15, 80%); and agreed that their participation increased their knowledge of trial reporting guidelines (11/15, 73%). Table 3 summarises the findings on feedback received in two themes, *what worked well* and *what could be improved*, which are described below. The two themes show that the PPIE was a cycle of learning for the implementers.

**Table 3:** Summary of what worked well and what could be improved for PPIE in the Delphi survey and consensus meeting

What worked well?	What could be improved?
**Learning workshop**	
• Content on trial reporting guidelines, surrogate outcomes, and Delphi survey • Engaging and interactive • Opportunity to learn from the lived experiences of other PPIE partners. • Reading resources after workshop	• Longer workshop to achieve a better understanding of complex issues around surrogate endpoints • Discussion of surrogate outcomes in other healthcare areas • Experiential learning with application of examples • Explain the importance of reporting guidelines to patients and the public
**Supports during Delphi survey participation**	
• Glossary of terminology • Help text on items being rated. • Clear instructions on how to rate items	
**Financial compensation**	
• Providing financial compensation for PPIE activities	• Clarity on expectations on need for documents as proof of bank details beforehand
**Consensus meeting**	
• Opportunity for PPIE partners to contribute first	• Use of better technology so that virtual participants can have a similar experience to that of physical participants*

*Not specific to PPIE, but a general suggestion for hybrid consensus meetings

### What worked well?

Most PPIE partners who attended the learning workshop perceived it as helpful. Sixteen (out of 19) partners took part in a survey after the workshop and before the Delphi survey, and 15 took part in another survey after completion of the Delphi survey. The workshop was felt to increase attendees’ confidence in trial methodology research (13/16, 81%), surrogate outcomes (16/16, 100%) and e-Delphi surveys (13/16, 81%). This was further reflected in feedback survey free-text comments and emails, which generally complemented the workshop’s organisation, content, engaging and interactive nature, and opportunity to learn from lived experiences from participants of trials that had used surrogate outcomes. Additionally, 87% of PPIE partners (*n* = 13/15) reported using reading resources shared after the workshop. Further, for those who participated in the Delphi survey (*n* = 13), the majority felt that the following features were helpful: glossary of terminology (11/13, 85%); help text on items being rated (13/13, 100%); and clear instructions on how to rate items (12/13, 92%). Overall, 73% (*n* = 11/15) of workshop attendees felt they had a clear understanding of the survey and had the support needed to participate, while 67% (*n* = 10/15) stated that they had enough information to contribute to the survey. Additionally, one PPIE partner mentioned financial compensation as a strength of the PPIE in the project. At the hybrid consensus meeting, PPIE partners felt it was well organised and valuable for the chairs to give PPIE partners the opportunity to speak before other delegates. Despite hybrid meetings having the particular challenge in that during lively in-person group discussion, the remote members can be overlooked, having one partner (DS) in the meeting room was valuable, as throughout the meeting, he actively sought contributions from remote PPIE contributors, interrupting the discussion in the room if necessary.

### What could be improved?

Despite the success of the learning workshop, there was room for improvement. Some attendees felt that workshop content would have been improved through (1) extending discussion of surrogate outcomes to other health areas such as maternal health (examples during the workshop were on cancer, cardiovascular diseases, and HIV); (2) experiential learning by providing examples for attendees to apply knowledge imparted; (3) more time explaining what reporting guidelines are and how their use could be important for patients and the public. Furthermore, after the workshop, some PPIE partners raised concerns about receiving a request for financial documentation to facilitate payment. One PPIE partner mentioned these concerns as a reason for not taking part in the e-Delphi survey. Finally, although not specific to PPIE, one partner felt the need for better organisation of hybrid consensus meetings, especially on technology, to enable virtual participants to have an almost similar experience to in-person participants.

### Reflections

#### PPIE partners’ reflection

This reflection was collectively written by the four PPIE partner co-authors (DS, AW, SM, RH).

Involvement in this innovative piece of work has been a stimulating, participatory, and learning journey for us, albeit hampered only by connectivity issues during virtual meetings for some. Our awareness of surrogate endpoint methodology has been expanded, and we are now far more empowered to make meaningful PPIE contributions to the use and reporting of this methodology in clinical trials.

As PPIE contributors, we brought our own unique life experience to the discussions, but also, importantly, acted as ‘Everyman’. Putting ourselves in the shoes of others was both necessary and useful to illustrate possible scenarios and to help clarify points in the discussions. Stepping back sometimes and asking, ‘How is this helpful for the patient?’, helped in the discussions.

We would want to emphasise the cooperative, collegial feel to the project, setting the right tone, which was well done and was absolutely key to success. This was especially important in the hybrid consensus meeting, as we rarely felt excluded, except when the technology let us down with poorly balanced sound. It’s so difficult to do everything well with such a large group.

As lay people, this was a wonderful opportunity to learn from significant experts. We feel very privileged to have been part of the discussions and to have one-on-ones with several people. This is one of those incidental benefits of working in this way: it may not be a core outcome of the project, but in whatever learning activity we think it is important not to lose sight of the incidental benefits of a project like this, conducted in this way. Culture is nurtured and advanced, and attachment to the common task is enhanced.

#### Researchers’ reflection

This reflection was written by two researcher co-authors (AMM, RST) and edited by all other researcher co-authors.

From the onset, we acknowledged that PPIE was vital in our project as the use of surrogate outcomes, if not properly reported or interpreted, can negatively impact patients’ health outcomes. Therefore, we set out to actualise PPIE by having DS lead in implementation with our full support. DS attended all project team meetings, and his contributions and questions were always refreshing and thought-provoking. He reminded and nudged us to consider what mattered to patients and where the use and better reporting of surrogate outcomes in trials would fit in this. Apart from the patient voice, DS contributed to ideas on other project activities such as participant mobilisation. In addition to the formal project meetings, we (AMM, RST) met DS on informal virtual meetings to plan PPIE activities, which strengthened our working relationship.

A key highlight of the project was organising a learning workshop to onboard the project to PPIE partners and build their capacity and confidence to be involved in the e-Delphi survey. Our target for the survey and learning workshop was 20 PPIE partners. We were doubtful if we would manage to reach that number, and even if we did for the workshop, we were not sure if attendees would participate in the survey. However, the workshop turned out to be a great success with 19 PPIE partners (thanks to great mobilisation by DS, AW, and SM), and 87% of them reported having participated in the e-Delphi survey. Key components of this workshop were the significant contribution of PPIE partners (SM, AW, RH) in helping with the organisation of the workshop and the great facilitation by DS.

When selecting Consensus Meeting delegates, we invited five PPIE partners who took part in the e-Delphi survey, and four were able to join the meeting. Having the PPIE partners in the meeting was highly valuable. The partners eloquently presented the patient perspective and reminded delegates of why the project was important. Additionally, they helped with clarification of issues, corrected some grammar, suggested strategies to disseminate and implement the extensions, and created humour. This shows PPIE contributed more than just the patient perspective in the meeting. Finally, looking back, we learned a lot from the partners, including how to do PPIE better in such projects as outlined in the experiences section above.

## Recommendations

Table 4 summarises the recommendations for PPIE in reporting guidelines based on our process, experiences, and reflections. We recommend starting involvement early: right from project conceptualisation and grant application. It is important to have a plan of involvement, which should be layered, i.e., involving PPIE partners in different phases of the project. This involvement should be meaningful, genuine, and authentic. Additionally, we recommend creating safe spaces and supporting partners in PPIE in ways which could involve organising learning workshops to onboard the project. Furthermore, involvement should be reflective, evaluated, and PPIE partners should be informed of the project’s progress. Finally, we recommend having a budget to implement PPIE and provide fair compensation for involvement.

**Table 4:** Recommendations for PPIE in trial reporting guidelines based on the SPIRIT|CONSORT-SURROGATE project

Recommendation	Explanation	Values* demonstrated
Involve early	• Start involvement at the project conceptualisation and grant writing phase • Allow for realistic timeframes for involvement	• Respect
Involve with a plan and layered approach	• Have a plan on how you will involve partners in various project activities but be flexible in this involvement. • Consider having a PPIE steering committee (3–5 partners) who can help with implementation of the plan. • Involve in various project phases, e.g., Delphi survey, consensus meeting, knowledge translation	• Support • Respect • Transparency • Responsiveness • Fairness of opportunity • Accountability
Involve meaningfully in a genuine way	• Consider what will be helpful involvement in the project to avoid tokenism while having some control over the involvement • Methodology projects offer PPIE partners and researchers mutual opportunities to gain from each other’s experiences and knowledge to inform and learn about the research	• Respect
Involve with support and in safe spaces	• Consider organising a learning workshop or engagement initiatives to introduce the project, including subject matter and what is expected in involvement. • Learning or engagement initiatives should be safe spaces which encourage interaction and engagement. • Have various support features, such as help texts and glossaries, to aid in participation in the project activities, such as Delphi surveys. • Be flexible on the support you give and how you organise your engagement initiatives	• Support • Fairness of opportunity
Involve with evaluation, reflection, and feedback	• Reflect on how involvement was undertaken, what went well, and what can be improved. • Consider sharing your reflections with other researchers. • Keep PPIE partners informed on the progress of the project	• Respect • Transparency • Responsiveness • Accountability
Involve with a budget	• Have a budget for compensation and other costs that come with involvement†. • Simplify the payment process or be clear about what is expected for payment to be made	• Support

*The values listed are based on INVOLVE’s Public involvement in research: values and principles framework [43]

†For useful references, see [41, 42]

## Discussion

### Summary of findings and recommendations

This article documents the process and experiences of PPIE in the SPIRIT and CONSORT-Surrogate project and draws recommendations for future projects. PPIE started at the project conceptualisation stage with a PPIE partner involved in the funding application and proceeded to the development of a strategy outlining PPIE integration in the project phases. Based on the experiences, we make six recommendations for integrating PPIE in reporting guidelines projects: involve early; involve with a plan and layered approach; involve meaningfully in a genuine way; involve with support and in safe spaces; involve with reflection and feedback; and involve with a budget (see Table 4). The successful integration of PPIE in our project can be summarised as having three key contributors: the PPIE strategy, the people involved, and positive relationships. We discuss each of these contributors further.

### PPIE strategy

The PPIE strategy helped define and structure involvement in the project phases. This is consistent with other PPIE reflections, which reported planning of involvement as contributing to meaningful involvement and avoiding tokenism or involvement as an afterthought [43–45]. To ensure implementation of developed PPIE strategies, dedicated time and resources are needed [43–47]. In our project, funding for PPIE was included in the grant application and supplemented by savings made from using a cheaper survey platform. Funders are increasingly requiring PPIE to be included in funded projects [48], providing researchers with an opportunity to include PPIE costs in grant applications. However, even when PPIE costs are covered, the size of the grant matters, with large programme and trial grants able to develop and sustain PPIE infrastructure and activities to a greater extent than small grants and fellowships [45]. Furthermore, even getting funding for methodology research projects, including the development of trial reporting guidelines [49], can be challenging, which makes cost a key barrier to implementing PPIE in such projects [50]. Nevertheless, we recommend that researchers involved in trial reporting guidelines consider the feasibility of PPIE within their available resources and alternative or efficient ways to implement involvement. For example, recruiting partners through online platforms, holding a virtual PPIE learning workshop and a hybrid consensus meeting meant that our costs were greatly reduced. Finally, researchers and partners should endeavour to evaluate and reflect on involvement after project completion as part of the PPIE strategy. Our evaluation and reflection found that PPIE was largely positive and valuable to both partners and researchers, but also identified areas of improvement, such as a longer learning workshop. Documenting, monitoring, evaluation, and reflection on PPIE is reported in literature as an important step to audit the impact of involvement, identify areas of improvement and best practices for future projects [43, 45–47]. In sum, a PPIE plan can promote meaningful involvement; the plan should be funded or implemented in the most efficient way possible and evaluated after project completion.

### People involved

Three groups of ‘people’ contributed to the success of PPIE in our project: PPIE partners, PPIE advisory group members, and researchers. Nineteen PPIE partners participated in the e-Delphi survey, denoting successful involvement given that Delphi surveys are key steps in the development of reporting guidelines. To build the capacity and confidence of partners, a 2-hour virtual learning workshop was organised, and the majority who attended the workshop participated in the e-Delphi survey. Training, induction sessions, and knowledge exchange are key requirements for building confidence for involvement, meaningful involvement and contribution to the decision-making process, as reported in the wider PPIE literature [43, 44, 46, 47, 51]. A smaller group of partners who were members of the PPIE advisory group was involved in (1) the learning workshop (refining the workshop structure and learning materials, helping with recruitment of participants, and encouraging contribution from peers); (2) the consensus meeting (contributing to discussions and bringing the patient and public perspective); and (3) dissemination (drafting of a section of the final manuscripts). Similarly, reflecting on PPIE in the Asthma UK Centre for Applied Research as an exemplar, Jackson et al. found a Patient Advisory Group to be a key component of PPIE organisational infrastructure, which improved recruitment for PPIE in projects and fostered involvement from project conceptualisation to dissemination [45]. Finally, researchers in the project acknowledged the vital role of meaningful PPIE, given the limitations of using surrogate endpoints in trials and the need for more engagement of patients and the public in such trials. Researchers’ attitudes towards PPIE are key to successful involvement in research projects [52]. Based on our experiences, we recommend that trial reporting guidelines project teams establish a PPIE steering or advisory group to help guide and facilitate wider involvement in the project. Furthermore, such projects should consider involving PPIE partners in all project activities and particularly key decision-making activities – Delphi survey and consensus meeting. The success of involvement is dependent on researchers’ willingness to involve PPIE partners and acknowledge the value of such involvement.

### Positive relationships

A positive relationship between partners and researchers was another key component of PPIE in our project. The relationship was fostered by practising INVOLVE values: respect, support, transparency, responsiveness, fairness of opportunity and accountability [39]. For example, in our project, researchers respected PPIE partners’ knowledge and experiences and involved them during project funding application and team constitution, e-Delphi survey to rate the importance of items, consensus meeting to finalise items, and knowledge translation, including helping with writing sections of final manuscripts. On the other hand, partners worked well with other project contributors, respecting their knowledge and building on their expertise. Responsiveness was shown by: researchers allowing partners to voice their opinion, including having a PPIE partner as the consensus meeting co-chair, and giving the first opportunity to speak to partners; partners agreeing to be involved in various activities within given timelines – Table 4 shows how other values map on different involvement recommendations. Similar to our experience, Dawson et al., in reflecting on PPIE in doctoral research, noted that positive relationships built on all six INVOLVE values were key to PPIE during and beyond the project [53]. Additionally, Burke et al. argue that positive relationships based on trust may be even more important for PPIE in methodology research, given that it is less familiar to partners than clinical research [54].

## Strengths and limitations

This article adds to the modest literature to date on PPIE in methodology research, particularly outlining best practices for PPIE in the development of trial reporting guidelines. This project shows that our approach is feasible, as was shown in a recent report on the development of a reporting guideline for systematic literature reviews of outcome measurement instruments, in which PPIE had a positive impact both on the content and presentation of the guideline [23]. Nevertheless, the experiences described in the present paper could also be considered in the context of limitations. Recruitment of PPIE partners involved a convenience sample, the majority of whom were UK-based with prior knowledge of trials, rather than a random and international sample of PPIE partners. This may limit the generalisability of findings and promote gatekeeping of PPIE opportunities to those with prior knowledge of trials. Secondly, due to time constraints, PPIE was not integrated into the literature review phase of the project, and this could have resulted in missing out on insights that were not in published records included in the literature review. However, the PPIE lead (DS) co-authored the literature review, and the review identified a PPIE-related item on informing participants on the use of surrogate endpoints, which was included in the guidelines [12, 13]. Thirdly, the development and piloting of the e-Delphi survey did not involve PPIE partners, which could have improved the participation experience of partners.

## Conclusion

This article describes the successful integration of PPIE in the development of SPIRIT and CONSORT-Surrogate reporting guidelines, providing recommendations for trial methodology projects. Involvement should start at the project conceptualisation stage; be formalised using an involvement strategy or plan and a budget; be conducted in a meaningful way supported by positive relationships between researchers and PPIE partners; and with reflection and evaluation to identify lessons and best practice.

## Supplementary Information

Below is the link to the electronic supplementary material.

Supplementary Material 1

Supplementary Material 2

Supplementary Material 3

## Data Availability

All project data can be accessed through the UK Data Service Reshare [55].
