# Supplementary material for: Patient and public involvement and engagement in methodology research: process, experiences, and recommendations from the SPIRIT- and CONSORT-Surrogate project
Source: Res Involv Engagem. 2025 Dec 4;11:144. doi: 10.1186/s40900-025-00807-y (PMC12720433; doi:10.1186/s40900-025-00807-y)
Supplement: Supplementary file 1 — Supplementary Material 1 [file 40900_2025_807_MOESM1_ESM.docx]

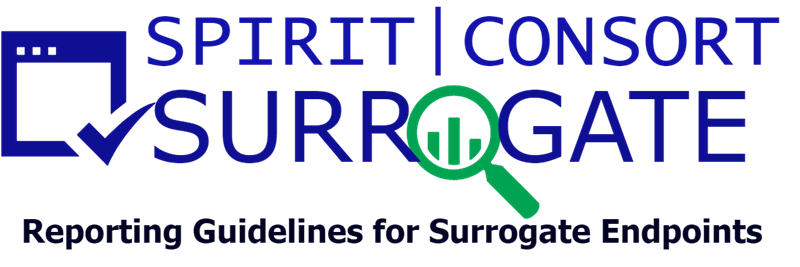


Surveys used

Confidence Survey

An informal and entirely unscientific line in the sand

* Required

**Some background**

This survey invites you to consider your levels of confidence with this research. It will help us understand how to better prepare for the workshop and gain information as to whether it has been helpful.

We will invite you to repeat the survey at the end of the workshop and ask whether you might be willing to revisit it in a few months time.

You may want to make a note or save a copy for your own benefit.

**Do you feel the workshop increased your level of confidence with:**

1. Patient, public involvement with **Health Services** in general *

| None | Fair | Moderate | Reasonable | Good | Strong |
| --- | --- | --- | --- | --- | --- |

| 2. Patient, public involvement with **Health Research** * |  |  |  |
| --- | --- | --- | --- |
| None Fair Moderate | Reasonable | Good | Strong |

| 3. Patient, public involvement with different **Research Methods** * |  |  |
| --- | --- | --- |
| None Fair Moderate Reasonable | Good | Strong |

| 4. Patient, public involvement with **Trials Methodology Research** * |  |  |
| --- | --- | --- |
| None Fair Moderate Reasonable | Good | Strong |

|  | 5. Patient, public involvement with **Surrogate Outcome Endpoints** * |  |  |
| --- | --- | --- | --- |
|  | None Fair Moderate Reasonable | Good | Strong |

| 6. Patient, public involvement with **Delphi survey** * |  |  |  |
| --- | --- | --- | --- |
| None Fair Moderate | Reasonable | Good | Strong |

7. Do you have any thoughts about the mutual benefits from this relationship between researchers and public/patient partners with trials methodology research?

This content is neither created nor endorsed by Microsoft. The data you submit will be sent to the form owner.


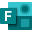


Microsoft Forms

Post-workshop Confidence Survey

* Required

Section

This survey invites you to consider your levels of confidence after attending the workshop. It will help us gain information as to whether the workshop was helpful and what we can learn from it for future projects.

You may want to make a note or save a copy for your own benefit.

**Do you feel the workshop increased your level of confidence with:**

1. Trials Methodology Research *

Yes

No

1. Surrogate Outcomes/Endpoints *

Yes

No

1. Delphi Studies *

Yes

No

1. Do you have any thoughts or comments about the workshop?

This content is neither created nor endorsed by Microsoft. The data you submit will be sent to the form owner.


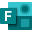


Microsoft Forms

2/2

Post-Delphi survey

This survey invites you to evaluate what facilitated or hindered your participation in the Delphi survey and your evaluation of the PPI engagement initiative. Your responses will be very important in informing how we can better engage patients and the public in projects seeking to develop reporting guidelines.

* Required

1. Did you take part in the Delphi survey? *[Required]* *

Yes both Round 1 and 2

Yes Round 1

Yes Round 2

No

1. **Support for participation in the Delphi survey**

Please indicate your level of agreement with each of the statements below *[Required]*  *

Neither agree nor

Strongly disagree Disagree disagree Strongly agree Agree

I had a clear understanding of the purpose of the survey

The supports I needed to

participate were available

I had enough information to contribute to

the survey

1. What else supported your participation in the survey? *[Optional]*
2. If you did **NOT participate in the survey completely, or in Round 2 of the survey**, what hindered you from participating? *[Optional but highly encouraged if it applies to you]*
3. **Impact of learning workshop**

Please rate how HELPFUL the learning workshop was with: *[Optional]*

No help Little help Some help Much help

Understanding about surrogate outcomes

Taking part in the Delphi

survey

1. Did you use the reading resources provided at the end of the project information sheet after the workshop? *[Required]* *

Yes

No

1. **Delphi participation experience**

If you took part in the Delphi, how useful was the following for enhancing your participation experience *[Required if you participated in Delphi]*

No help Little help Some help Much help

Glossary of

Terminology

Help text on items

Instructions on rating items

# Final thoughts

Please provide your final thoughts on the PPI engagement initiative in this project through participation in the learning workshop and/or Delphi survey

8. As a result of my participation in this project, I am better informed about trial reporting guidelines *[Required]* *

| Strongly disagree | Disagree | Neither agree or disagree | Agree | Strongly agree |
| --- | --- | --- | --- | --- |

| 9. Overall I was satisfied with this engagement initiative *[Required]* * |  |  |
| --- | --- | --- |
| Neither agree or  Strongly disagree Disagree disagree | Agree | Strongly agree |

| 10. This engagement initiative was good use of my time *[Required]* * |  |  |
| --- | --- | --- |
| Neither agree or  Strongly disagree Disagree disagree | Agree | Strongly agree |

1. What were the strengths of this engagement initiative? *[Optional]*
2. What could be improved about future engagements for projects developing reporting guidelines? *[Optional]*
3. What else would you like us to know about your experience with this engagement initiative? *[Optional]*
4. Please provide an email address so as to receive your gift voucher

This content is neither created nor endorsed by Microsoft. The data you submit will be sent to the form owner.


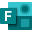


Microsoft Forms
