# Supplementary material for: Patient and public involvement and engagement in methodology research: process, experiences, and recommendations from the SPIRIT- and CONSORT-Surrogate project
Source: Res Involv Engagem. 2025 Dec 4;11:144. doi: 10.1186/s40900-025-00807-y (PMC12720433; doi:10.1186/s40900-025-00807-y)
Supplement: Supplementary file 3 — Supplementary Material 3 [file 40900_2025_807_MOESM3_ESM.docx]

**Development of a SPIRIT extension (SPIRIT-**

**SURROGATE) and CONSORT extension**

**(CONSORT-SURROGATE)**

Patient and Public Involvement Strategy

Version 1, 24/03/2022

**Prepared by (on behalf of the Project Management Group)**

Name: Role: PPI Lead

Signature: Date:

Name: Role: Project Manager

Signature: Date:

| **1.** | **Project Summary** |
| --- | --- |

The SPIRIT|CONSORT-SURROGATE project^^[[1]](#footnote-1)^^ aims to develop and disseminate

CONSORT (Consolidated Standards of Reporting Trials)^^[[2]](#footnote-2)^^ and SPIRIT (Standard Protocol Items: Recommendations for Interventional Trials)^^[[3]](#footnote-3)^^ extensions to improve reporting of trials and protocols with surrogate primary endpoints. The project will have four phases: Phase 1 (Literature reviews) to identify items to be included in extensions and a subset of participants for a Delphi study; Phase 2 (Delphi study) to rate the items identified in Phase 1; Phase 3 (Consensus meeting) to agree on final items for inclusion in extensions; and Phase 4 (Knowledge translation) to disseminate the project outputs, through various strategies such as publications, key partners, and engage stakeholders to improve implementation of developed extensions.

We aim to integrate patient and public involvement (PPI) in all the project phases as described in this strategy. The implementation of the strategy will be led by the PPI Lead (XX) and project manager (XX), and supported by Principal Investigators (XX), and Co-investigators (XX).

| **2.** | **Implementation activities** |
| --- | --- |

# 2.1 Inception meeting with PPI representatives

We will identify 3-5 PPI representatives who will contribute to: identifying other PPI contributors to participate in the Delphi study; and commenting on items identified from the scoping review. These representatives will be invited to a virtual meeting where the project will be introduced, the proposed PPI activities

presented, and any proposed changes noted. Prior to this meeting, a briefing sheet of useful background information, to read or just be familiar with, will be sent, see Appendix 1. We will aim to have this meeting by mid-May 2022.

# 2.2 Consultation on items from the scoping review

After completion of data synthesis from the scoping review (Phase 1), the 3-5 PPI representatives who attended the inception meeting will be consulted (one hour virtual meeting) on the identified items that will be rated in the Delphi study. They will comment on language, relevance and offer any additional items not identified in the review. We will aim to have this meeting in late June/early July.

# 2.3 Learning Workshop on surrogate endpoints

To facilitate meaningful participation in the Delphi study (Phase 2), we will invite about 20 PPI representatives for a 2-hour training on RCT design and surrogate endpoints. The invite is with an ‘expectation’ of participation or perhaps people using wider networks to encourage completion in the Delphi study. We will consider having a pre-meet/drop-in session before the workshop to clarify on practical issues of the training to participants. The structure of the workshop will be as follows:

- Start: Welcome by Facilitators
- About the workshop and clarifying PPI role
- Randomised controlled trials – use, design
- Surrogate endpoints – what, why, how, limitations
- Questions
- About the project
- Some background about SPIRIT/CONSORT Guidelines
- Questions in smaller groups
- About the Delphi Study – What, when and how
- End of workshop

# 2.4 E-Delphi survey

The 20 PPI representatives who will be trained will be encouraged to participate in the e-Delphi survey. Other PPI representatives will also be invited to register and participate in the survey.

# 2.5 Consensus Meeting

We will select 2-4 PPI representatives who have completed the Delphi study, to participate in making the final decision of the items to be included in extensions (Phase 3). The consensus meeting will be held in March 2023.

# 2.5 Knowledge Translation

We will inform all Delphi participants, including PPI representatives, on the progress of the project after completion of the Delphi study including when the final extensions have been published. We will explore disseminating the extensions and reflections on PPI integration in the project through various ways such as organising a webinar, writing a blog or a peer reviewed publication. These activities will start after finalisation of extensions, tentatively from May 2023.

# 2.6 PPI Debrief

To evaluate, reflect and learn from our experience of PPI in the project, we will follow up on email on people’s experiences participating in the project and potentially invite about 10 PPI representatives for a virtual one-hour project debrief session. This session will be recorded (with participants consent) to allow for detailed meeting notes to be taken. These notes will inform writing of reflective pieces/publications on PPI involvement in the project. The debrief is likely to be held in April 2023. Throughout the project, researchers and PPI contributors will be encouraged to take reflective notes on their learning and experiences that will be discussed in this debrief.

| **3.** | **PPI budget cost** |
| --- | --- |

We will offer payment to PPI representatives for their involvement in the following activities: inception meeting, consultation on scoping review findings, learning workshop, and PPI debrief. Those who attend the learning workshop will be paid one hour for pre and post reading. We will not offer payment for participation in data collection activities: Delphi study and consensus meeting. The NIHR payment guidance^^[[4]](#footnote-4)^^ for members of the public will be used. Additionally, we will offer certificates of participation to people who attend the learning workshops and complete the Delphi study. Table 1 shows the proposed budget.

**Table 1: Project PPI implementation budget**

| **Activity** | **Maximum**  **participants** | **Rate in £** | **Total** |
| --- | --- | --- | --- |
| Inception meeting | 5 | 25 | 125 |
| Consultation meeting on scoping review findings | 5 | 25 | 125 |
| Learning workshop | 20 | 75 | 1500 |
| PPI debrief | 10 | 25 | 250 |
| **Total** |  |  | **2000** |

# Appendix 1


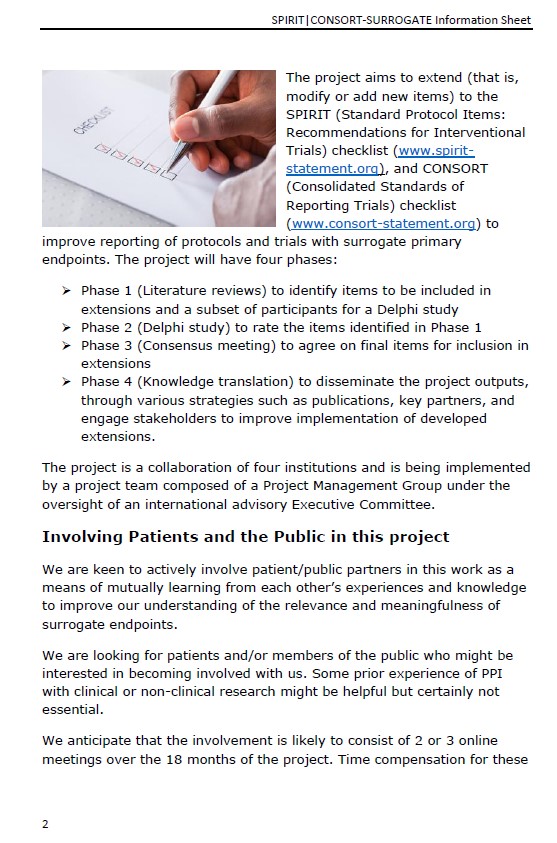


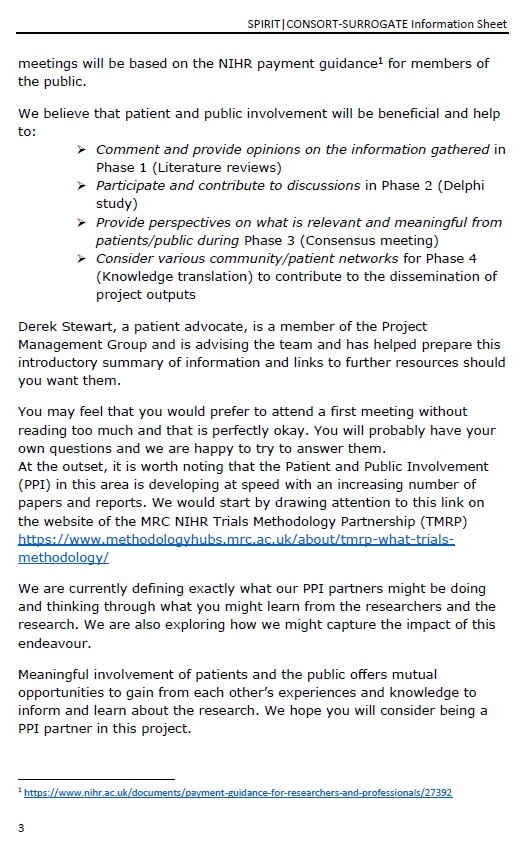


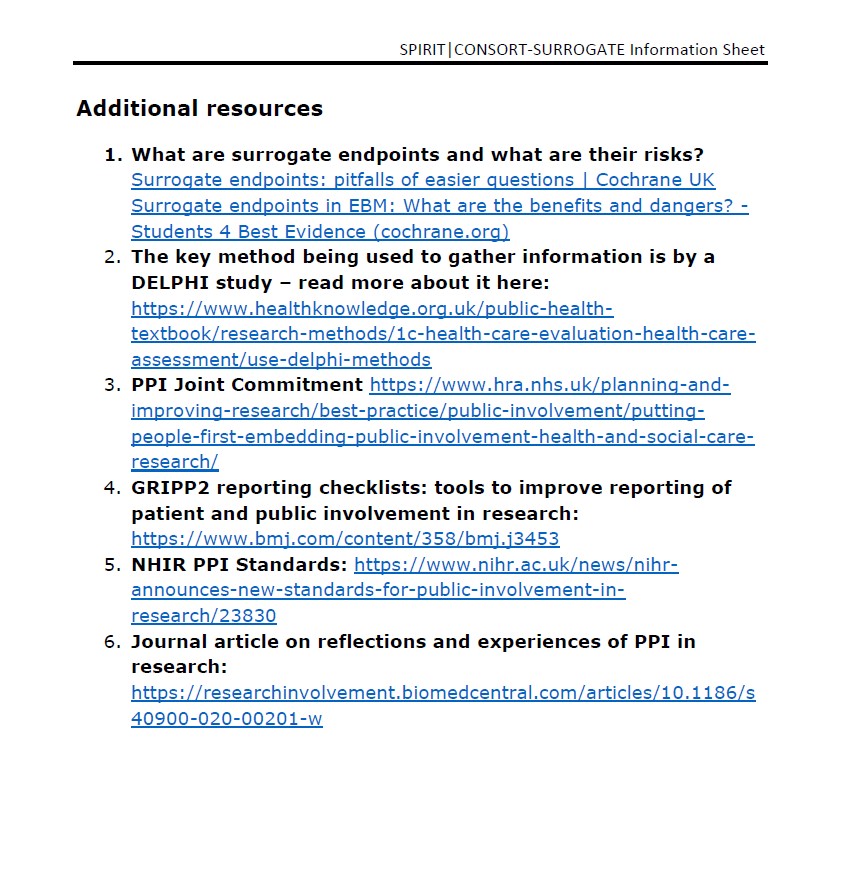


1. See the website page for summary on the project [https://www.gla.ac.uk/researchinstitutes/healthwellbeing/research/mrccsosocialandpublichealthsciencesunit /programmes/complexity/methods-development/spirit-consort-surrogate/](https://www.gla.ac.uk/researchinstitutes/healthwellbeing/research/mrccsosocialandpublichealthsciencesunit/programmes/complexity/methods-development/spirit-consort-surrogate/)

   [↑](#footnote-ref-1)
2. <http://www.consort-statement.org/>

   [↑](#footnote-ref-2)
3. <https://www.spirit-statement.org/>

   [↑](#footnote-ref-3)
4. <https://www.nihr.ac.uk/documents/payment-guidance-for-researchers-and-professionals/27392>

   [↑](#footnote-ref-4)
